# Impact of liver resection on portal venous pressure and renal function

**DOI:** 10.1186/cc12342

**Published:** 2013-03-19

**Authors:** P Biesenbach, T Grünberger, B Payer, A Ferlitsch, W Druml, E Fleischmann, F Luf, J Mühlbacher

**Affiliations:** 1 Medical University of Vienna, Austria

## Introduction

Liver dysfunction - in correlation to the severity of functional impairment - but also any increase in portal pressure *per se *(hepatorenal reflex) can induce alterations in renal function and ultimately result in hepatorenal syndrome (HRS). In this prospective investigation we determined the impact of liver resection on portal venous pressure by measuring the hepatic venous pressure gradient (HVPG), on concentrations of vasoactive peptides and on renal function.

## Methods

Twenty patients (mean age 66.3 years) undergoing elective liver resection surgery because of malignant tumor were assessed and grouped according to resection size: (Group 1) hemihepatectomy, *n *= 13 fiversus (Group 2) segmentectomy, *n *= 7. HPVG was measured before and after resection by canulation of a hepatic vein under fluoroscopic guidance, liver function was assessed by the indocyanine green plasma disappearance rate (ICG-PDR).

## Results

HVPG increased in Group 1 from 3.7 to 5.4 mmHg (*P *0.05) and decreased in Group 2 (4.8 to 4.3 mmHg, *P *= NS) (Table 1). Liver function as assessed by ICG-PDR decreased in Group 1 by day 1 (18.5 to 15.3, *P *0.05) and remained stable in Group 2 (25.5 to 26.8, *P *= NS). Renin, aldosterone, ADH, adrenaline, noradrenaline and dopamine increased significantly (*P *0.05) in Group 1 during operation. Group 2 showed a significant rise only in ADH and dopamine. Acute kidney injury occurred in five of 13 patients in Group 1 (*P *0.05).

**Table 1:** 

	Group 1	Group 2
Renin	36.4	7.9
PostOP	189.7*	30.5
Day 1	258	31.3
ADH3.49	2.59	
PostOP	16.11*	10.8*
Day 1	10.8	2.39
Creatine	0.8	0.83
Day 1	1.25*	0.91

*P <0.05 compared with preOP value.

## Conclusion

Depending on resection size liver resection acutely increases portal venous pressure and induces neurohumoral activation resulting in compromised renal function and increased risk of developing AKI.

